# Intimin and Invasin Export Their C-Terminus to the Bacterial Cell Surface Using an Inverse Mechanism Compared to Classical Autotransport

**DOI:** 10.1371/journal.pone.0047069

**Published:** 2012-10-09

**Authors:** Philipp Oberhettinger, Monika Schütz, Jack C. Leo, Nadja Heinz, Jürgen Berger, Ingo B. Autenrieth, Dirk Linke

**Affiliations:** 1 Institut für Medizinische Mikrobiologie und Hygiene, Universität Tübingen, Tübingen, Germany; 2 Abteilung 1, Max Planck Institut für Entwicklungsbiologie, Tübingen, Germany; University of Helsinki, Finland

## Abstract

Invasin and intimin are major virulence factors of enteropathogenic *Yersiniae* and *Escherichia coli*, mediating invasion into and intimate adherence to host cells, respectively. Several studies have hinted that extracellular portion of these homologous proteins might be exported *via* an autotransport mechanism, but rigorous experimental proof has been lacking. Here, we present a topology model for invasin and intimin, consistent with the hypothesis that the N-terminal β-barrel domain acts as a translocation pore to secrete the C-terminal passenger domain. We confirmed this topology model by inserting epitope tags into the loops of the β-barrel. We further show that obstructing the pore of β-barrel hinders the export of the passenger domain. As for classical autotransport, the biogenesis of invasin and intimin is dependent on the Bam complex and the periplasmic chaperone SurA, whereas the chaperone/protease DegP is involved in quality control. However, compared to classical autotransporters (Type Va secretion), the domain structure of intimin and invasin is inverted. We conclude that proteins of the intimin and invasin family constitute a novel group of autotransported proteins, and propose that this class of autotransporters be termed Type Ve secretion.

## Introduction

Autotransporters are proteins of Gram-negative bacteria that can transport large domains of their own polypeptide chain to the bacterial cell surface; this transport is mediated by a transmembrane domain that resides in the outer membrane. The transported part of the protein is usually referred to as the passenger domain. Several classes, named type Va to Vc secretion systems, have been described in detail [1]. The classical, monomeric autotransporters (type Va) comprise such important pathogenicity factors as Antigen 43 of *Escherichia coli* (*E. coli*) [2], or IgA1 protease or NalP of *Neisseria meningitidis* [3], [4]. Type Vb secretion systems are two-partner systems typically organized in operons, where one protein constitutes the transporter, while the second protein is transported through the outer membrane [5]. The Type Vc secretion system comprises the trimeric autotransporters that export three polypeptide chains through a trimeric pore [6]. Recently, an autotransporter family composed of a patatin-like domain, a POTRA domain, and an autotransporter domain was found and termed type Vd secretion [7].

From our bioinformatics efforts to better understand the evolution of transmembrane β-barrel proteins [8], we came to the conclusion that the pathogenicity factors intimin of *E. coli* and invasin of enteropathogenic *Yersinia spp.* are members of the same β-barrel protein class.

Intimin is an important virulence factor of enteropathogenic *E. coli* (EPEC) and mediates intimate adhesion to the intestinal epithelium of the host. The receptor for intimin binding is Tir, a protein that is translocated into the host cell membrane by the bacterium itself [9]. Invasin is produced by the enteropathogenic strains *Yersinia enterocolitica* and *Y. pseudotuberculosis* and mediates host cell attachment *via* high-affinity binding to ß1-integrins [10]. In the past, the adhesive activity of both proteins, mediated by their C-terminal effector domains, has been investigated in detail. In addition, the structure of the invasin (of *Y. pseudotuberculosis*) and the intimin C-terminal effector domains were resolved years ago [11], [12]. Both proteins are potent virulence factors and may well serve as potential targets to be exploited for disease prevention and/or treatment. Nevertheless, there is only little reported interest in understanding the mechanism of their biogenesis despite the fact that this knowledge may also lead to the identification of promising drug targets.

It has been suggested that invasin and intimin are autotransporters analogous to monomeric (Type Va) autotransporters [13]. No experimental proof was presented, except indirectly by the fact that heterologous domains can be expressed on the cell surface [14], [15] comparable to autodisplay systems developed from classical (Type Va) autotransporters [16]. Consequently, we have recently proposed that intimin and invasin be included in Type V secretion classification, and suggested naming this group of autotransporters Type Ve secretion [17]. In this work, we present a model for intimin and invasin based on thorough sequence analysis and, using insertions of epitope tags into the loops and turns of the β-barrel transmembrane domains, show that the topology of intimin and invasin is consistent with our model. As the C-terminus of these proteins is exported, the mechanism must be inverted compared to Type Va secretion and is thus fundamentally different. This is also reflected by the different types of passenger domains secreted by these proteins, which are immunoglobulin (Ig) domains, not β-helices. We thus present experimental validation for the novel Type Ve secretion mechanism.

## Results

### Bioinformatics

**Figure 1:**
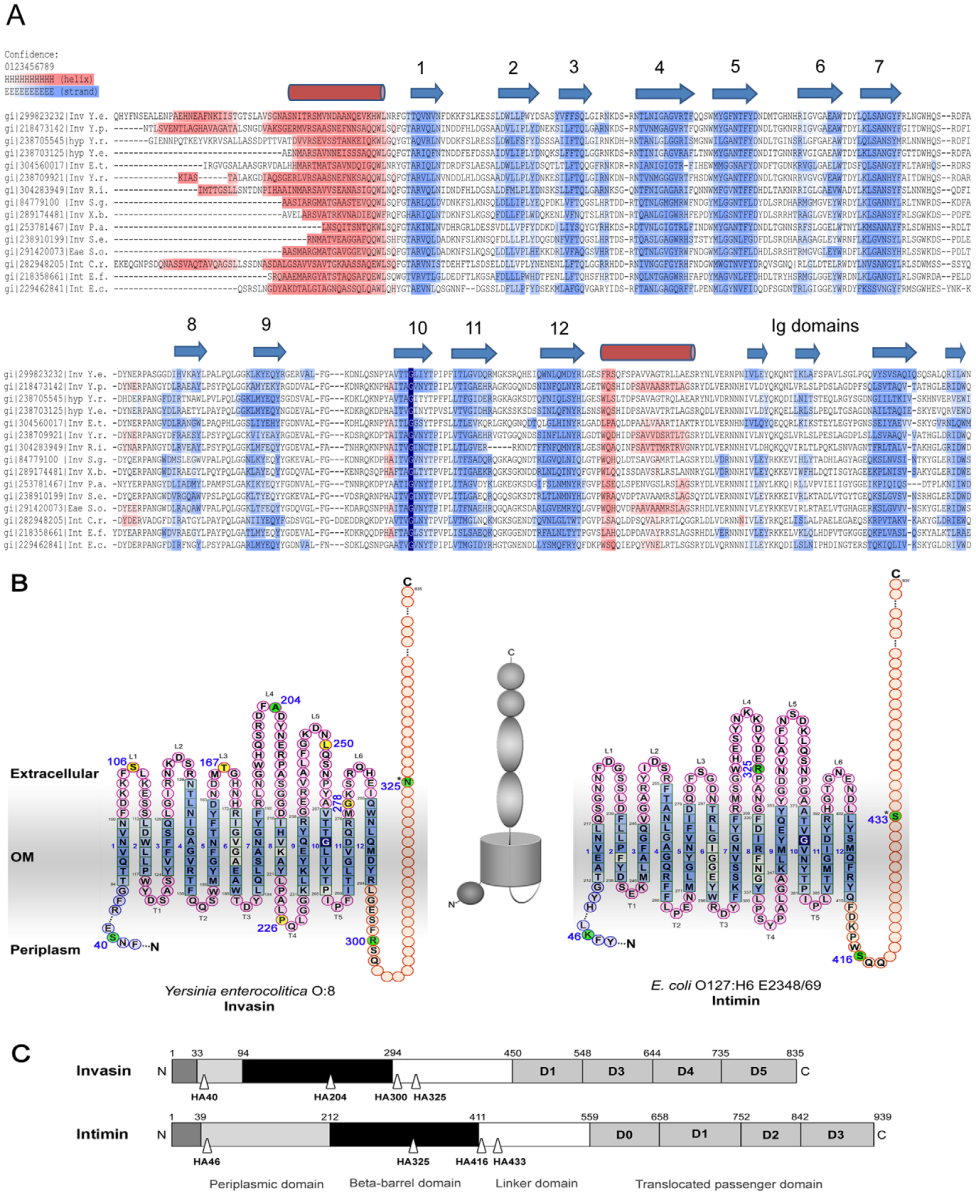
Schematic domain organisation and topology models of *Yersinia enterocolitica* O:8 invasin and *E. coli* O127:H6 E2348/69 intimin. (**A**) Domain predictions. The amino acid sequences of the N-terminal part of each protein (excluding the extracellular passenger domain) were analysed using SignalP, PsiBLAST and HHAlign. α-helical segments (magenta) and β-strands (blue) are indicated by different colouring. The conserved glycine residue (see text) is indicated in dark blue. A scalable version of the alignment with more sequences can be found in Fig. S1. The position of the weakly predicted autotransport helix following β-strand 12 is inferred from the consensus of all sequences (for discussion, see text). (**B**) Topology models derived from our bioinformatical predictions are shown. β-strands are coloured blue, the interconnecting loops (extracellular) and turns (periplasmic) are drawn in pink. The α-helical linker and the C-terminal passenger domain are shown in orange. Residues after which two HA tags were introduced are coloured yellow (introduction of the tag disturbed protein biogenesis) or green (protein was produced and properly inserted into the OM) and the respective position is indicated. (S  =  strand, L  =  loop, T  =  turn). A schematic figure is shown in between the topology models. In the mature protein the C-terminal passenger is threaded through the N-terminal barrel and the pore is closed by the linker adjacent to the N-terminus of the passenger. (**C**) Domain organisation and location of HA tag insertions. The individual domains of invasin and intimin are depicted. Sites of HA tag insertions are labelled by triangles. The aa positions after which tags were introduced are indicated.

The basis of our analysis was the finding that intimin and invasin are part of the same beta-barrel protein family as defined by the database HHOmp [8] and suggested by other authors [13], [18]. The similarity of the extracellular Ig domains was already known [12], [19]; we set out to understand the similarities (or differences) of the transmembrane domain, based on the assumption that both proteins must be autotransporters [13]. After compiling an alignment of intimin and invasin homologues that includes secondary structure predictions (Figure 1A), we found that all proteins of the family consist of an N-terminal small helical periplasmic domain that presumably binds peptidoglycan [20], and the well-known C-terminal extracellular part [12], [21], which flank a central transmembrane β-barrel domain with 12 β-strands. This domain is linked to the exported Ig-domains by a linker, which we hypothesize is analogous to the α-helical linker observed in the structures of classical type Va autotransporters such as NalP [22]. Thus, the helical linker should occlude the autotransport-competent pore after successful autotransport; however, in contrast to type Va autotransporters, it is not the N- but the C-terminus which is transported. This implies a completely inverted mechanism – a novel class of autotransporters. The autotransport function may explain the relatively low confidence of the helix propensity prediction observed in Figure 1A. The helix is not only composed of small residues due to space constraints in the pore - it must also be flexible to adopt different, non-helical conformations during the transport process before it folds into its final structure. Figure 1B shows the topology models deduced from the alignment and from the secondary structure predictions. The membrane-embedded parts of the proteins consist of twelve β-strands, which are connected by extracellular loops (L1–L6) outside and periplasmic turns (T1–T5) inside the cell. To determine if the topology model and the autotransport hypothesis are correct, we performed different experiments as described below, all based on this topology model.

### Experimental Proof of the Correct Domain Border Prediction

**Figure 2:**
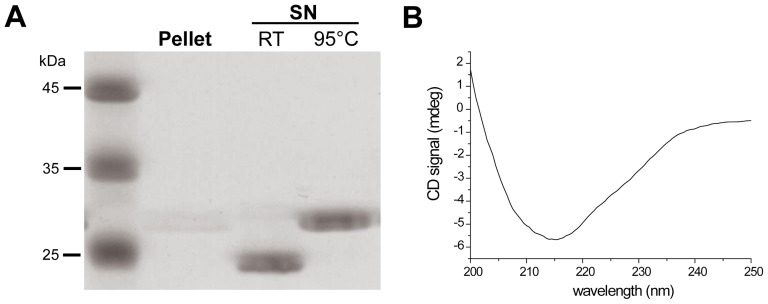
Refolding of invasin membrane anchor (InvMA). **(A)**Heat-modifiability of refolded InvMA. Guanidine-denatured, recombinant InvMA (expected molecular weight  = 29 kDa) was refolded by rapid dilution into 1% LDAO. The majority of the protein remains in the supernatant (SN) after centrifugation. Heating refolded InvMA at 95°C in the presence of urea leads to slower migration in SDS-PAGE compared to an unheated sample (RT  =  room temperature). The faster migration of the unheated sample is typical of folded β-barrel membrane proteins. (**B**) CD spectrum of refolded InvMA in LDAO buffer. The spectrum is typical of a β-structured protein with the characteristic broad minimum at 215 nm, showing that InvMA is folded.

Domains in proteins are defined as autonomously folding units; these units can then be combined into larger proteins. In the case of invasin and intimin, the extracellular domains are known. To show that our predictions are correct, we cloned the transmembrane fragment, *i.e.* the 12 predicted β-strands plus the α-helical linker. The protein was produced as inclusion bodies, and was therefore misfolded from the start. We purified the protein in a denatured state using guanidine-containing buffer systems combined with Ni-NTA chromatography. We then checked whether the protein fragment could fold autonomously. β-barrel proteins are known to refold *in vitro* in detergent-containing buffer systems (*e.g.* [23]). We screened numerous detergents and buffer conditions, varying also the ionic strength and the pH of the buffers. As β-barrel proteins are not easily denatured in sodium dodecyl sulfate (SDS) sample buffer, folding of these proteins can be shown in SDS-polyacrylamide gel electerophoresis (PAGE) where the samples are not heated prior to running the gels. The proteins run at a different apparent molecular weight compared to heated (denatured) samples. This effect is called heat modifiability or electrophoretic mobility shift [24]. Figure 2A shows such a mobility shift for the transmembrane domain of invasin, after using 1% lauryldimethylamine-N-oxide (LDAO) as the detergent for refolding. Samples were centrifuged after refolding; the pellet contains almost no protein, indicating that the protein folded into a detergent-soluble form instead of aggregating. The same soluble sample, when assayed with circular dichroism (CD) spectroscopy, displays a clear β-strand signal with a negative peak at ∼ 215 nm (Figure 2B). These observations clearly show that we predicted the domain borders correctly, and that the cloned fragment is a transmembrane domain that can fold independently.

### Insertion of Epitope Tags

To further experimentally verify our topology models for intimin and invasin, we introduced epitope tags into the predicted (i) loops and turns, (ii) into the small helical periplasmic domain at its very N-terminus, (iii) the α-helical linker between the β-barrel and the passenger domain and (iiii) the C-terminal passenger domains. We introduced different epitope (myc-, FLAG- and HA-) tags as single tags or tandem arrays to test if they interfere with invasin and intimin production. Based on these preliminary results we decided to use a double HA epitope tag linked by the flexible amino acid triplett GSG, yielding a GSG-HA-GSG-HA-GSG double tag. This tag enabled us to yield sufficient signal to detect it by immunofluorescence stainings and flow cytometry. Throughout the manuscript this double HA epitope tag will be referred to as HA tag. Initially, all mutants containing the HA tags were created with the invasin protein. Owing to the direct correlation of flexibility with the length of a loop or turn, we presumed that the biogenesis and folding of the β-barrel should be disturbed to a lesser extent if the HA tag was introduced into the longer loops of the barrel. Hence, we started by inserting the HA tag directly into the middle of loop 4 (L4) after A204 of invasin (Fig. 1B). As invasin HA204 was produced and also inserted into the outer membrane we next tried to tag loops L1, L3, L5 and L6. These mutants copurified with OM preparations and were detectable by western blots which exhibited reduced protein levels compared to the wild-type protein. Due to the limited sensitivity we were not able to detect these mutants by immunofluorescence or flow cytometry. Thus, they were excluded from our analyses. The insertion of the HA tag into the predicted longest periplasmic turn (T4) after F226 of invasin and also the extension of the extracellular L3 to the length of L4 with a prolonged flexible linker significantly impaired membrane insertion and thus these mutants were not suitable for further analysis. Furthermore, we decided to introduce an epitope tag into the small helical periplasmic N-terminal domain (at site S40) and into the α-helical linker at the C-terminus of the beta-barrel (at site R300), which resulted in successful membrane integration. In addition, insertion of HA tags after N325 combined with a deletion of the C-terminal Ig domains (Δ326–835) allowed proper β-barrel assembly in the outer membrane and detection of the tag. Finally, we ended up with a set of 4 HA-tagged variants of invasin and the corresponding variants of intimin (Fig. 1B). In the following, we analyzed all of these mutants for outer membrane insertion, passenger domain translocation and binding affinity to host cells in detail and checked whether the mutant proteins support our topology models for invasin and intimin.

### Insertion of HA Tags into Invasin and Intimin at Specific Positions does not Interfere with OM Insertion and β-barrel Assembly

**Figure 3:**
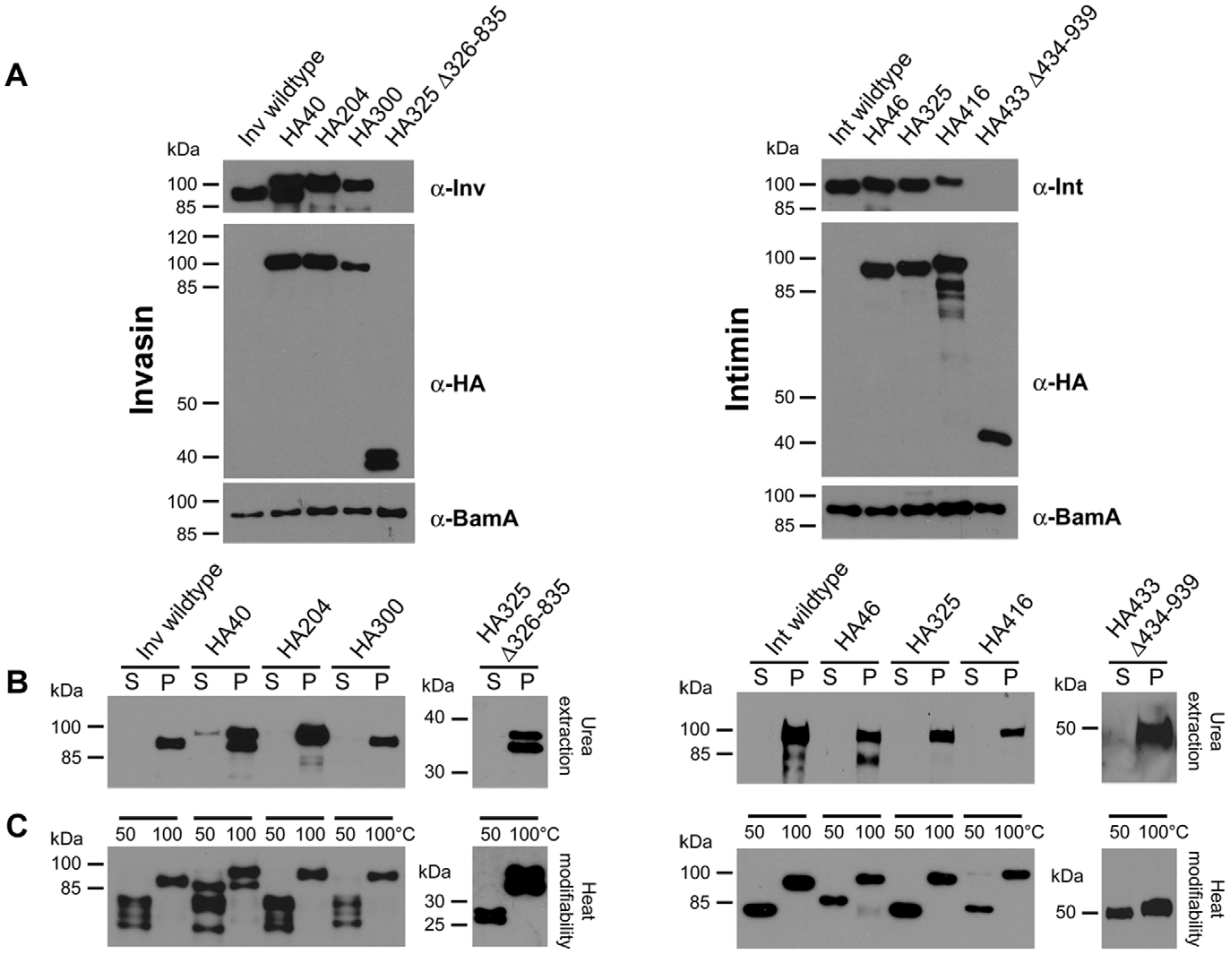
Expression, stable outer membrane integration and heat modifiability of wild-type invasin and intimin and the HA-tagged variants thereof. (**A**) Invasin/intimin protein and HA-tagged variants are expressed and can be detected in OM preparations. Expression of invasin/intimin was induced by addition of AHTC in an exponentially growing culture of *E. coli* omp2 and outer membranes were prepared. All protein variants could be detected with invasin/intimin or HA tag antibodies. BamA was used as a loading control. (**B**) Urea extraction. To test proper OM insertion, the preparations were treated with urea and the insoluble membrane integrated protein fraction (P  =  pellet) and soluble proteins (S  =  supernatant; not stably integrated into the OM) were separated and analysed by Western blot. Invasin/intimin were detected with anti-InvA, anti-EaeA or anti-HA antibodies (applies only for the truncated variants Inv HA 325 Δ326–835 and Int HA 433 Δ434–939). (**C**) Heat modifiability is a feature of β-barrel proteins and indicates proper folding of the β-barrel domain. Electrophoretic mobility after incubation at 50°C compared to 100°C of wild-type invasin and intimin protein and all variants was analysed by SDS-PAGE and Western blot.

The generation of invasin and intimin protein variants containing affinity tags in order to assess the topology of the barrel within the outer membrane yielded a set of 4 proteins each for invasin and intimin (Inv HA40, Inv HA204, Inv HA300, Inv HA325 Δ326–835 and Int HA46, Int HA325, Int HA416, Int HA433 Δ434–939). In order to examine if the proteins were produced and integrated into the bacterial outer membrane, membrane fractions were analysed by Western blotting with antibodies directed against invasin, intimin or the HA tag (Fig. 3). The tagged versions of invasin and intimin were detectable in the outer membrane fraction either with invasin- or intimin-specific antibodies (wild-type proteins and all mutants except the truncated ones; Fig 3A and B left side, upper panel) and also HA tag antibodies (except wild-type invasin and intimin; Fig 3A and B left side, middle panel). To demonstrate equal loading, we probed the outer membrane fractions also with an antibody detecting the β-barrel assembly machinery component BamA (Fig. 3 left side, lower panel). Thus, all proteins co-purify with OM preparations.

To rule out that the Western blot signals resulted from protein that was not properly integrated into the OM but only loosely attached, we extracted the outer membrane fractions with 6 M urea. After extraction, properly inserted protein should remain in the pellet fraction, whereas all other proteins are found in the supernatant. Western blots with both fractions clearly indicate that all proteins tested were pellet-associated and only a negligible amount of invasin or intimin was solubilized by the urea treatment (Fig. 3A and B right side, panels: urea extraction). Thus, all the produced protein variants are stably integrated into the outer membrane. In order to find out if the insertion of HA tags somehow interfered with the β-barrel folding of invasin or intimin, we next determined the heat-modifiablity of all our proteins. This feature is characteristic for β-barrel proteins [24]. Upon heating invasin or intimin in Laemmli buffer at 95°C, the running behaviour in SDS-PAGE is altered if the barrel is correctly folded. Denatured β-barrel proteins tend to migrate differently from their not-fully denatured counterparts that were only heated to 50°C. Figure 3 (A and B, right side, panels: heat modifiability) clearly demonstrates that the heat modifiability of HA-tagged invasin and intimin is comparable to that of the corresponding wild-type proteins. Taken together, we demonstrated that all tagged constructs behave like the wild-type proteins: they co-purify with the outer membrane, they are all stably integrated into the OM and that their β-barrels display heat-modifiability (though the shift is not so pronounced for intimin HA433 Δ434–939; however, shifts for different constructs can exhibit variable behavior [23]). Thus, we could use the tagged invasin and intimin variants to analyse their topology and biogenesis.

### Immunofluorescence Stainings Support the Predicted Localisation of HA Tags in Invasin and Intimin

**Figure 4:**
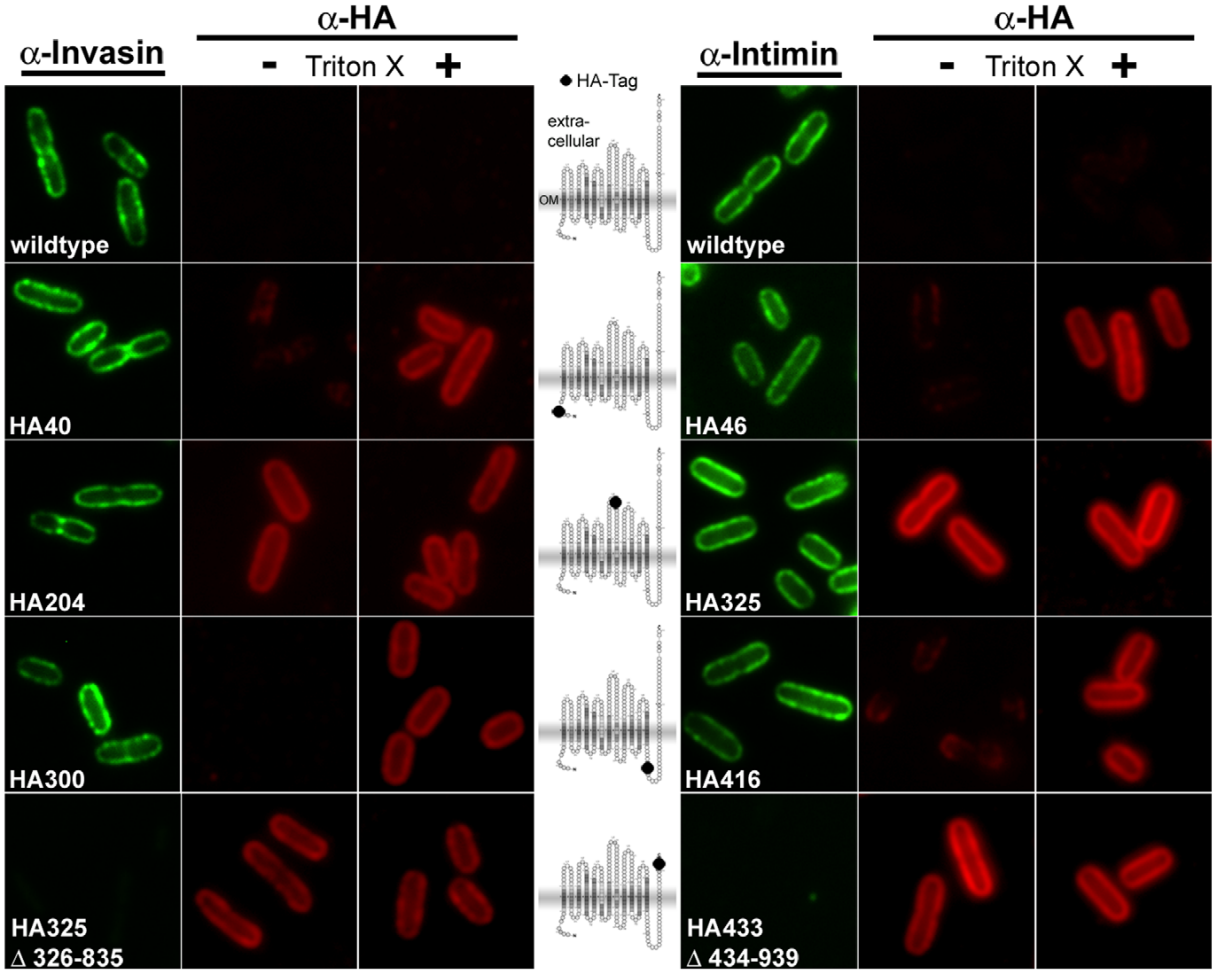
Immunofluorescence staining of invasin and intimin wild-type and HA-tagged variants expressed in *E. coli* omp2. Bacteria were incubated with the primary antibody (anti-Inv, anti-EaeA or anti-HA). For detection of periplasmic protein the cells were permeabilized, as only epitopes that are exposed on the bacterial surface can be detected in non-permeabilized cells. Each sample was probed with antibodies directed against the C-terminal part of the passenger domain (C-terminal 195 aa of invasin or 280 aa of intimin, respectively) and antibodies directed against the HA tag. Miniaturized invasin topology models with the insertion sites of the HA tag (black dots) are shown in the middle of the figure. As the predicted position of the labels also applies for intimin and for sake of clarity, we did only include the invasin miniature models in this figure.

In order to validate our predictions of the invasin and intimin topology, we analysed the subcellular localisation of the HA tags using immunofluorescence microscopy. Tags that reside within the periplasm should only be accessible after permeabilization of the cells. Tags that are located extracellularly should be accessible without treatment of the cells with detergent. To test this, cells producing wild-type invasin or intimin or one of the HA-tagged variants were fixed and either permeabilized or not before incubation with the first antibody directed against the HA affinity tag. As a control, a set of all cells was stained with specific antibodies for invasin or intimin (Fig. 4). All these control samples yielded a ring-shaped fluorescence signal associated with the membrane except Inv HA325 Δ326–835 and Int HA433 Δ434–939, respectively. The reason for this is that the specific antibodies for both invasin and intimin were raised against the very C-terminus of the proteins (the last 397 aa of invasin or the last 280 aa of intimin) and these binding epitopes are missing in the truncated mutants. Immunofluorescence stainings with the HA tag antibody without prior permeabilization resulted in a membrane-associated staining pattern in Inv HA204 and Int HA325, where the HA tag was predicted to reside in an extracellular loop connecting the beta-strands 7 and 8. The stainings of Inv HA325 Δ326–835/Int HA433 Δ434–939 where the HA tag was predicted to be exposed extracellularly at the truncated end of the translocated effector domain resulted in a signal associated with the membrane. In contrast, when we stained Inv HA40, Int HA46, Inv HA300 and Int HA416, where the HA tags were supposed to be located within the periplasm, no significant fluorescence signal could be detected without prior permeabilization. However, when we permeabilized the same cells prior to antibody incubation the staining resulted in a clear ring-shaped fluorescence signal. Thus, the immunofluorescence stainings confirmed all of our topology predictions.

### HA Tags do not Interfere with Effector Domain Translocation

**Figure 5:**
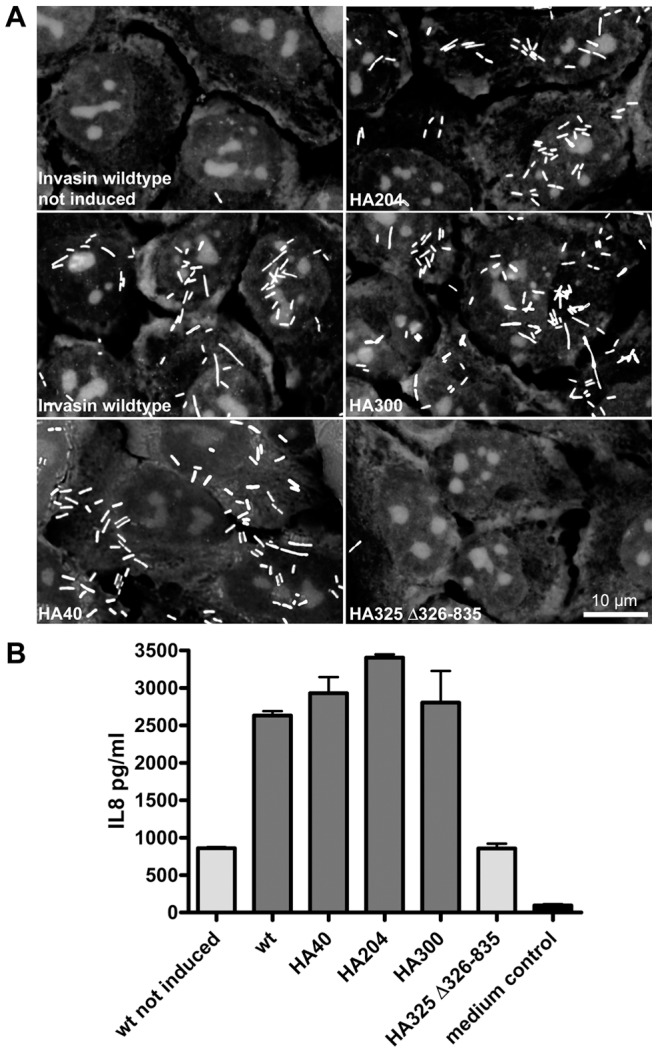
Adhesion behaviour of bacteria expressing invasin wild-type or HA-tagged variants and their ability to induce IL-8 secretion in HeLa cells. (**A**) Adhesion behaviour. Invasin producing *E. coli* strains were tested for their ability to mediate adhesion to HeLa cells. Bacteria were allowed to adhere to a HeLa monolayer grown on coverslips and imaged with a brightfield microscope. Only those bacteria producing invasin constructs that properly display their C-terminus on the bacterial surface can adhere. (**B**) Induction of IL8 secretion. Supernatants of HeLa cells that were stimulated with *E. coli* strains that produced invasin wild-type protein and HA-tagged variants thereof were assayed for the presence of IL-8. Only strains that properly expose their passenger domain on the bacterial surface are able to induce significant IL-8 secretion.

Both invasin and intimin mediate adhesion to host cells via their C-terminal effector domains. Whereas invasin binds directly to host cell integrins [25], intimin binds to the translocated intimin receptor Tir that is translocated into the host cells by the pathogen itself [9]. As we postulate that invasin and intimin are members of a new class of autotransporters that use their N-terminal beta-barrel to translocate their C-terminal effector domain we had to take care that the introduction of HA tags did not interfere with passenger domain translocation. Therefore, we carried out adhesion assays using our mutants and analysed the invasin-induced interleukin 8 (IL-8) secretion of HeLa cells using an enzyme-linked immunosorbent assay (ELISA). Inv HA40, Inv HA204, Inv HA300 mediated adherence to HeLa cells at wild-type levels and comparable numbers of bacteria were found to be attached on the cell surface. Only bacteria that did not produce invasin (uninduced wild-type invasin) or bacteria that produced Inv HA325 Δ326–835, where the Integrin-binding domain was deleted, failed to adhere to HeLa cells (Fig. 5A). Concurrently, significant levels of IL-8 in HeLa cell culture supernatants were only found after stimulation with bacteria producing wild-type invasin, Inv HA40, Inv HA204 or Inv HA300. Thus, insertion of the HA tag did not interfere with the translocation of the invasin effector domain or its ability to mediate adhesion to and induce IL-8 secretion in HeLa cells in any of the HA-tagged invasin derivatives.

**Figure 6:**
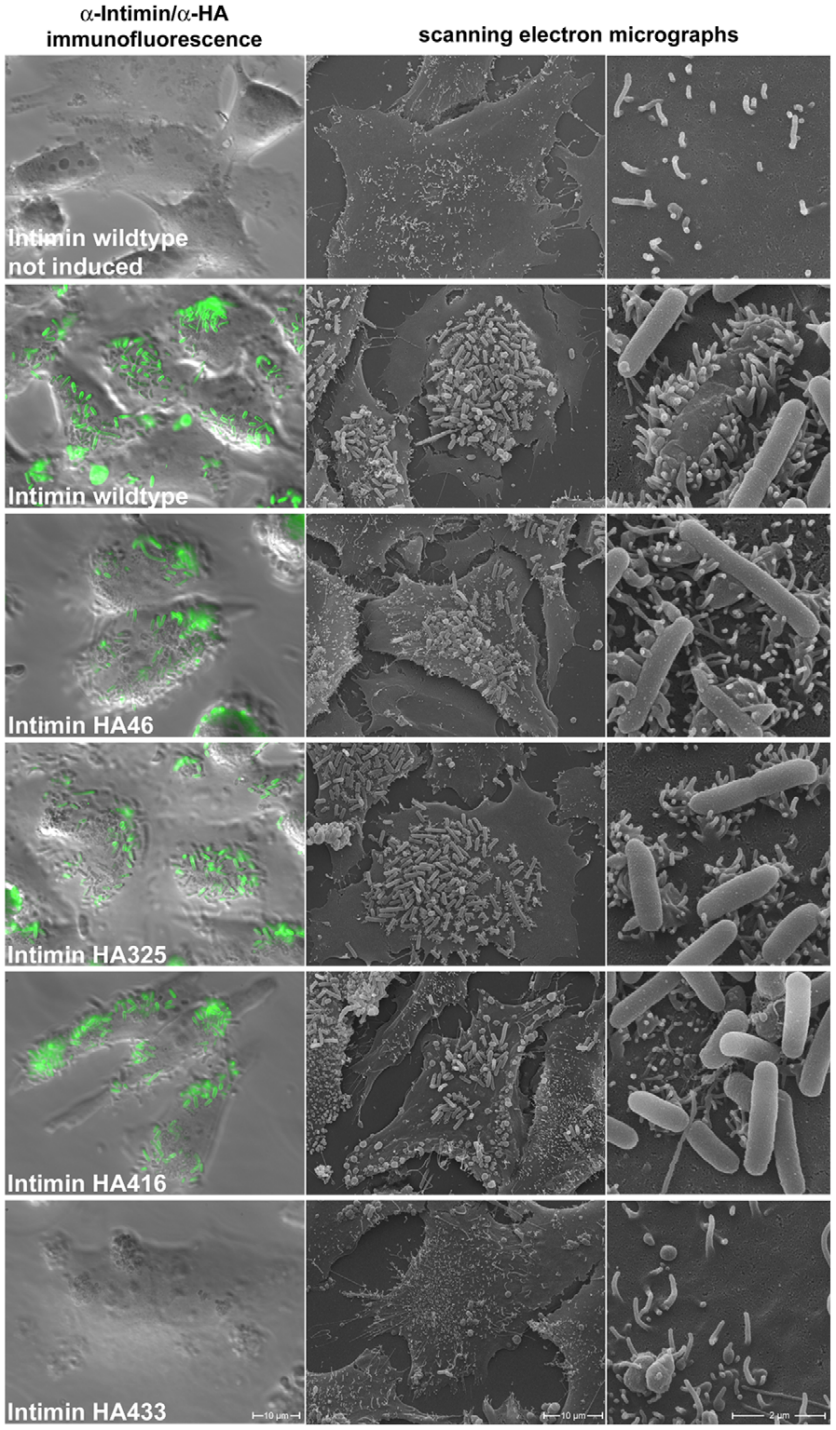
Adhesion of intimin-producing *E. coli* strains to preinfected HeLa cells carrying the Tir receptor. Adherent bacteria (green fluorescence) were stained with antibodies directed against intimin or the HA tag (applies for IntHA433; this construct is not recognized by the intimin antibody). Overlays of bacteria and differential interference contrast images are shown in the left column. The middle and right columns show scanning electron microscopy (SEM) images of corresponding samples. Numerous attached bacteria in various stages of invasion can be seen in those samples where intimin variants are produced that are properly inserted into and translocated across the OM.

To test the adhesion properties of intimin-producing bacteria we carried out a preinfection adhesion assay as described in detail in Materials and Methods. Adhesion was analysed by immunofluorescence microscopy stainings and the reorganization of the host cell cytoskeleton was monitored by scanning electron microscopy (Fig. 6; we also provide a faux-color scanning electron micrograph of bacteria producing wild-type intimin adhering to preinfected HeLa cells as a Striking Image). In order to discern the intimin-producing bacteria from possibly residual adhering EPEC *ΔeaeA*, we stained the bacteria with antibodies directed against intimin or the HA tag (Int HA433 Δ434–939) and secondary fluorescently labelled antibodies. The adhesion behaviour we finally observed resembled our observations for the HA-tagged invasin variants. Bacteria producing Int HA46, Int HA325 or Int HA416 adhered to the cell surface of the HeLa cells in numbers comparable to that of the wild-type intimin producing bacteria. As seen for invasin, we could not observe adhesion of bacteria that were not induced for intimin production or bacteria that produced the truncated version Int HA433 Δ434–939, which lacks the Tir binding domain. Overall, the adhesion assays demonstrate that the introduction of HA tags did not hinder adhesion to the natural substrates of invasin and intimin. Thus, these experiments demonstrate that the effector domains were properly translocated despite the presence of the HA tags and could be used to further analyse biogenesis and topology of invasin and intimin.

### Mutation of a Highly Conserved Glycine within the β-barrel Domain Leads to Impaired Translocation of the Effector Domain of Invasin

In the last years, several groups claimed that the effector domains of members of the intimin/invasin protein family might be translocated in an autotransporter-like fashion [13], [15], [18], [20], [26], [27], but experimental evidence for this hypothesis is lacking or only indirect. To tackle this problem, we took a similar approach as for the trimeric autotransporter YadA. We have shown previously for YadA that upon exchange of a highly conserved glycine residue located within the β-barrel domain and facing the pore lumen, the translocation efficiency of the effector domain correlated negatively with the side chain size of the residues that were introduced instead of the conserved glycine [28]–[30]. Less YadA passenger domain was exposed on the cell surface with increasing side chain size.

**Figure 7:**
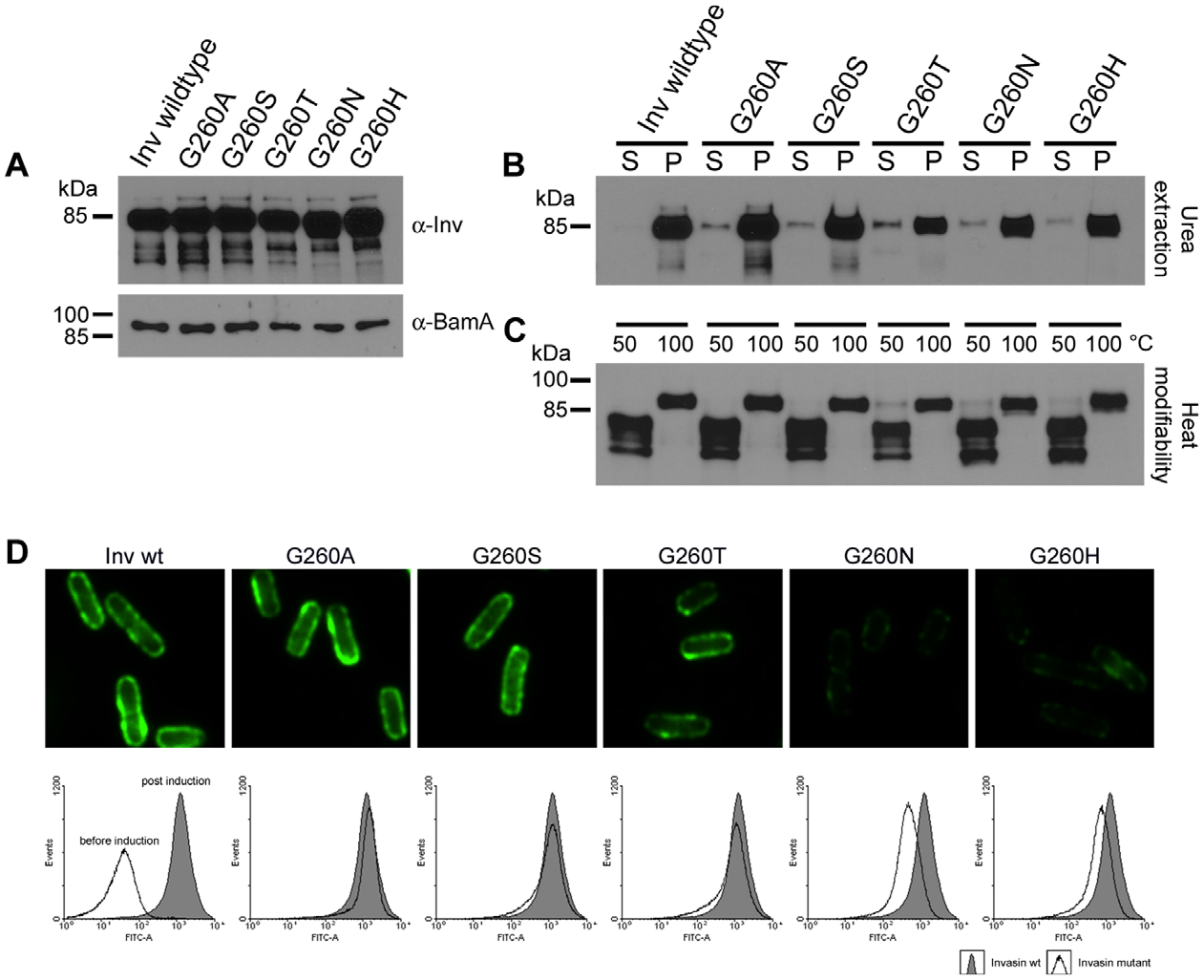
Substitution of a conserved glycine residue leads to impaired autotransport. A conserved glycine residue (G260) facing the pore lumen of the N-terminal beta-barrel was replaced by amino acids with increasing side chain size (A<S<T<N<H). (**A**) Production of the invasin variants was analysed by Western blot. All invasin variants are produced in comparable amounts and copurify with the membrane fraction. To demonstrate equal loading the samples were also probed with an antibody directed against BamA. (**B**) Stable integration of the invasin variants was verified by urea extraction of outer membrane preparations. Only residual amounts of protein can be found in the supernatant whereas the major fraction is stably associated with the outer membrane (S  =  supernatant, P  =  pellet fraction). (**C**) Heat modifiability of all samples indicates proper folding of the beta-barrels. (**D**) Autotransporter function of the beta-barrels was tested by measuring the outer membrane localisation of effector domains by immunofluorescence and flow cytometry. Histograms show overlays of invasin wild-type (grey filling) and the respective invasin G260 mutants (no filling).

The sequence alignments we used to develop our topology models for invasin and intimin (Fig. 1) revealed the presence of a highly conserved glycine residue. In invasin, G260 is predicted to be part of the tenth β-strand and thus of the N-terminal β-barrel. Consequently, we hypothesized that G260 may be important for passenger domain translocation in invasin and exchanged this glycine for amino acids of increasing side chain size (A<S<T<N<H) in order to block the transition of the passenger domain by narrowing the β-barrel pore diameter. Western blot analysis revealed that all variants (Inv G260A, G260S, G260T, G260N and G260H) are produced at wild-type levels and co-purify with membrane fractions (Fig. 7A).

To rule out that some variants are only weakly associated with the outer membrane we extracted the preparations with urea and tested the insoluble pellet fraction (P) and the soluble supernatant fraction (S) for the presence of invasin. As shown in Fig. 7B, the major fraction for all proteins harbouring a G260 amino acid exchange is found in the membrane pellet and only residual amounts are soluble in urea.

In order to find out if the exchange of G260 interferes with β-barrel formation, we tested the heat modifiability of all samples. All proteins exhibited the typical shift in running behaviour (Fig. 7C). Thus, we can safely assume proper β-barrel folding and membrane insertion. To test if the exchange of G260 interferes with passenger domain translocation, we performed immunofluorescence and flow cytometry analysis. As depicted in Fig. 7D both methods revealed a significantly decreased signal in those mutants where we exchanged G260 by larger amino acids (G260T, G260N and G260H).

Crosslinking experiments showed that the dimerisation of intimin is mediated by its β-barrel domain [20]. No dimerisation has been reported so far for the invasin β-barrel, but it is possible that also invasin dimerises *via* the transmembrane domain and that mutations in this region disrupt oligomerisation. To rule out that the reduced surface display of the invasin effector domain in the G260 mutants is due to an impaired capacity of the β-barrel domain to dimerise, we crosslinked wild-type invasin and the G260 mutants with BS3 (Bis [sulfosuccinimidyl] suberate) and used wild-type intimin as a control. Whereas intimin showed dimerisation after crosslinking, no crosslinked oligomers were observed for wild-type invasin or the G260 mutants (Fig. S2), as expected. We conclude that the passenger domain is translocated through the N-terminal β-barrel in an autotransporter-like fashion and that G260 plays an important role in facilitating this transport.

### The Biogenesis of Invasin Necessitates BamA and is SurA-dependent

It has been shown for classical autotransporters [31]–[34], for trimeric autotransporters [29], and recently also for intimin [26] that their biogenesis is assisted by periplasmic chaperones like SurA, Skp and DegP and depends on the presence of BamA. BamA is essential for growth of *E. coli*. We thus used a conditional depletion strain for our assays [29]. Moreover, we made use of individual *surA, skp* and *degP* null mutant strains from the Keio collection [35].

**Figure 8:**
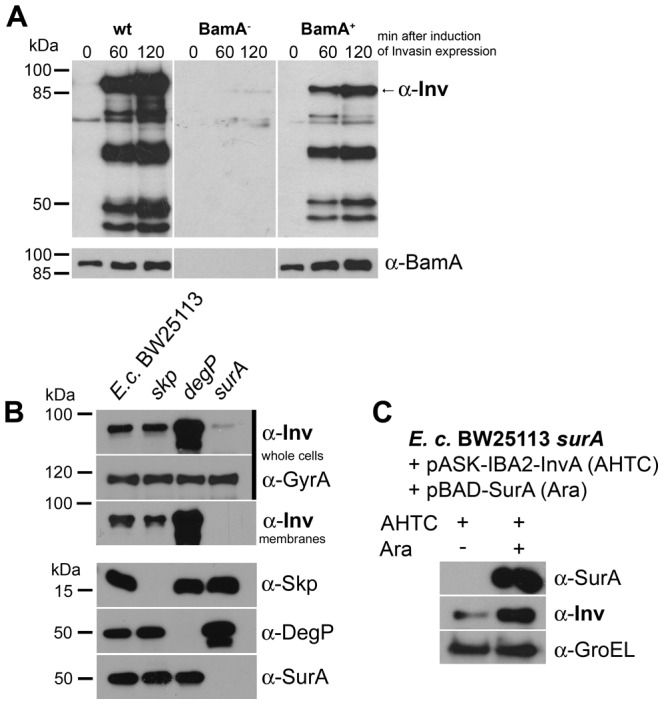
The biogenesis of invasin is BamA- and SurA- dependent. (**A**) Levels of invasin, BamA and DegP in whole-cell lysates. Bacteria were grown for 2 h under BamA depletion (–) or rescue (+) conditions, and then expression of invasin was induced. Whole-cell lysates were prepared 0, 60 and 120 min after the induction of invasin expression and analyzed by SDS-PAGE and western blotting using antibodies directed against invasin, BamA and DegP. (**B**) Effect of periplasmic chaperones on invasin levels in the outer membrane. Invasin protein levels in OM fractions of *skp, degP* and *surA* knockout strains and the corresponding wild-type *E. coli* were assessed 2 h after induction of invasin expression. Invasin, GyrA (loading control for whole-cell lysates), Skp, DegP and SurA protein were detected by Western blot analysis. The presence of invasin protein was tested in whole-cell lysates as well as in membrane fractions to demonstrate how the absence of specific chaperones influences membrane insertion of invasin. (**C**) Complementation of SurA. *E. coli* BW25113 Δ*surA* expressing invasin (+ AHTC) was grown in the presence (+) or absence (–) of arabinose for induction of *surA* transcription from pBAD28_*surA*. Whole-cell lysates were analyzed in SDS gels 2 h after induction of invasin expression and Western blots were immunodecorated with anti-SurA and anti-InvA as well as anti-GroEL as loading control.

As shown in figure 8A, the depletion of BamA resulted in a significant reduction of invasin protein after induction. However, when *bamA* expression was permitted, invasin expression levels were comparable to wild-type (Fig. 8A). Our data indicate that invasin is degraded in the absence of BamA. This is corroborated by the finding that, upon depletion of BamA (BamA^−^), DegP levels in whole-cell lysates are significantly elevated as compared to wild-type bacteria or under BamA rescue conditions (BamA^+^).

Next, we tested which chaperones are involved in invasin biogenesis. First, we verified the absence of SurA, Skp or DegP in the *E. coli* knockout strains by Western blotting. We were not able to detect the individual proteins in the knockout strains, but we observed an upregulation of DegP in the *surA* knockout strain as described by others (Fig. 8B, lower panel; [36]). Next, we transformed the verified *surA, skp* and *degP* null mutant strains and the corresponding wild-type strain with the invasin expression vector and assayed the presence of invasin after induction in whole-cell lysates and in outer membrane fractions. We did not observe differences in expression pattern of invasin in whole cells compared to the membrane fractions. Invasin levels were comparable to wild-type levels in the *skp* knockout strain but in the absence of DegP we observed a significant accumulation of invasin in the outer membrane. However, in the *surA* knockout strain invasin was no longer detectable in the membrane fraction (Fig. 8B). Complementation with a plasmid allowing expression of *surA* under the control of an arabinose-inducible promoter could recover wild-type invasin protein levels (Fig. 8C). Taken together, the biogenesis of invasin is BamA-and SurA-dependent. Moreover, DegP seems to be the major player in periplasmic quality control of invasin, whereas Skp is not essential.

## Discussion

Though several papers have suggested that intimin and invasin constitute a class of autotransported proteins [13], [18], experimental evidence to confirm the β-barrel domain and its topology has been lacking. We provide clear experimental evidence that intimin and invasin are indeed autotransporters and that they, together with the other members of the intimin/invasin family, represent a new class of autotransporters. The inverted domain order compared to classical, monomeric auototransporters, and the presence of a β-barrel transmembrane domain had long been recognized [13]; however, the membrane topology of these proteins was not established. We demonstrate that the β-barrel domain follows all rules for classical β-barrels, including heat modifiability, and membrane insertion through the Bam complex. This is in perfect agreement with the fact that all β-barrel proteins, including all autotransporters, are evolutionarily related [23], [37].

Introducing epitope tags into intimin and invasin did not work for all of the predicted loops and turns; the insertion efficiency of the resulting proteins was obviously impaired and most of the protein was degraded. Thus, detection of the corresponding passenger domains on the cell surface by immunofluorescence or flow cytometry failed. Nevertheless, as the functional tagged variants corroborate our topology predictions, we conclude that our suggested model is correct, and that the autotransport mechanism is delicate and may easily be disrupted.

Degradation of invasin was observed in a *surA* knockout mutant and upon depletion of BamA. We assume that this is due to accumulation of misfolded protein in the periplasm, which results in the induction of σ^E^ and Cpx envelope stress responses, upregulation of DegP production and the subsequent degradation of invasin within the periplasmic compartment [38]. This interpretation is also supported by the fact that in a DegP knockout strain we observed accumulation of invasin. Thus, it seems likely that DegP is the main factor within the periplasm responsible for the degradation of invasin. Similar findings have been presented for intimin [26].

The mechanism by which periplasmic chaperones and the Bam complex enforce the biogenesis of outer membrane proteins is still enigmatic. Our results suggest that the biogenesis of invasin, having an inverse domain order compared to classical autotransporters, shares at least some mechanistic details for insertion and assembly.

While this manuscript was in preparation, the group of Susan Buchanan published the crystal structures of the intimin and invasin β-barrels [39]. The structures (PDB IDs: 4E1S and 4E1T) are in excellent agreement with our topology predictions. The number of strands is the same, and, apart from a small shift for strands 2 and 3, the placement of the strands is almost identical. The one major difference is the linker region traversing the barrel. We had anticipated an α-helix within the pore, similar to classical autotransporters [22], but the crystal structures show a more extended conformation for the linker. Our predicted helical segment corresponds to an unexpected helical turn on the periplasmic side of the β-barrel. The linker, as shown by the structures, follows C-terminally to this helical region. The structure of the intimin β-barrel also provides a possible explanation as to why the intimin HA433 Δ434–939 mutant does not display heat modifiability: in our construct, the linking region (which we assumed was outside the barrel) is deleted, and therefore the stability of the β-barrel might be compromised as a number of charge-charge interactions between the linker and barrel wall are extirpated.

Autotransport is a process independent of ATP or a membrane potential as an energy source [40]. Despite significant efforts, the exact mechanism of classical (*i.e.* Type Va) autotransport remains unclear, However, most of the evidence points towards a hairpin model, in which the passenger is transported C-to-N-terminus through the pore of the beta-barrel domain [17]. For intimin and invasin, the β-barrel is N-terminal to the passenger, and therefore, if the hairpin model is correct, the passenger would be transported with an inverted polarity, *i.e.* N-to-C. We demonstrated that by introducing larger residues in the position of a conserved glycine in the invasin pore lumen, we can hinder autotransport. Whereas the translocator domain is still inserted into the OM, passenger domain translocation is hindered. The same effect is observed when mutating an equivalent glycine residue in a trimeric autotransporter [28]. In both cases, the most conserved residue is a glycine in the middle of the third β-strand counting from the C-terminus, and this is confirmed by the crystal structures [39]. The effects are somewhat less pronounced in invasin compared to YadA. The reason for this probably is that in a trimeric autotransporter such as YadA, the effect of a mutation is threefold (*i.e.* three positions in the transporter barrel are simultaneously modified). Based on our results and the crystal structures in particular, we now have a detailed understanding of the topology of the transmembrane β-barrels of the intimin-invasin family of bacterial adhesins. This will allow the rational design of mutagenesis and other experiments to further elucidate the mechanism of passenger secretion. With such knowledge to hand, a controlled disruption of autotransport by small molecules might lead to degradation of these important pathogenicity factors in the bacterial cell, a mechanism that would open the way to novel anti-infective drugs.

Considering the inverted domain order of intimin and invasin compared to classical autotransporters, sequential folding and transport of the passenger domain through the pore must proceed from N- to C-terminus of the passenger domain(s), and not from the C- to N-terminus as in *e.g*. pertactin or EspP [41], [42]. This difference in the transport mechanism tempts us and others [17], [18] to suggest that we are dealing with a new class of autotransporters. Following the previous nomenclature for type V secretion systems [1], and taking into consideration that another new type has been described recently as type Vd autotransporters [7], we propose to name the intimin/invasin family of autotransporters type Ve secretion systems.

## Materials and Methods

### Reagents, Chemicals, Antibiotics and Enzymes

If not stated otherwise, all reagents, chemicals and antibiotics were purchased from Applichem (Darmstadt, Germany) and restriction enzymes from Fermentas (St. Leon-Rot, Germany).

### Bioinformatics

The analysis of domain borders, secondary structure predictions, and alignments were calculated using tools in the environment of the MPI bioinformatics toolkit [43]. Homologues to intimin and invasin were found using PSI-Blast [44], and were verified against the HHOmp database [8]. The alignment was produced using MUSCLE [45] and the toolkit alignment viewer; secondary structure predictions were performed with Quick2D, a MPI toolkit applet that comprises multiple programs. The prediction of the domain borders, and the alignments, were verified using the profile-profile comparison tool HHPred [46].

### Cloning of *Yersinia* Invasin, EPEC Intimin and *E.coli surA*

The *invA* gene of *Yersinia enterocolitica* serotype O:8 and the *eaeA* gene of EPEC O127:H6 strain 2348/69 were amplified by PCR using chromosomal DNA as template. Primer sequences are available upon request. The PCR products were digested with HindIII and XbaI and cloned under the control of a tetracycline inducible promoter of pASK-IBA2 expression vector (IBA GmbH, Göttingen, Germany). The plasmids used in this study are summarized in Table 1. The PCR product of the *surA* gene was obtained from chromosomal DNA of *E.coli* K-12 BW25113 and cloned into the SacI and HindIII sites of pBAD28 [47] allowing *surA* transcription by the addition of arabinose. All mutants were verified by DNA sequencing.

**Table 1:** Strains and plasmids used in this study.

Name	Relevant genotype or description	Reference
***Yersinia strains***		
*Y.e.* WA-314	*Yersinia enterocolitica* WA-314 serotype O:8, clinical isolate, virulent wild-typestrain, pYV^+^, Nal^R^	[52]
***E. coli strains***		
*EPEC* O127:H6	enteropathogenic *E.coli* O127:H6 strain 2348/69	[53]
*E.c.* BL21(DE3)	F^-,^ *ompT hsdDB (r_B_^−^ m_B_^−^) gal dcm* (DE3)	Novagen
*E.c.* BL21(DE3)omp2	BL21(DE3), *ompF::Tn5*, Kan^R^	[54]
*E.c.* MC4100A	F’ *lacΔU169 araD139 rpsL150 thi flbB5301 deoC7 ptsF25 relA1 ara+*	[55]
*E.c.* MC4100A *bamA^−^*	*E.coli* BamA depletion strain, *bamA::kan*, P_BAD_ *::bamA, araC*, Kan^R^	[29]
*E.c.* wt K-12 BW25113	*rrnB3 ΔlacZ4787 hsdR514 Δ(araBAD)567 Δ(rhaBAD)568 rph-1*	Keio Collection, [35]
*E.c.* BW25113Δ*skp*	BW25113, *skp::kan*, Kan^R^	Keio Collection, [35]
*E.c.* BW25113Δ*surA*	BW25113, *surA::kan*, Kan^R^	Keio Collection, [35]
*E.c.* BW25113Δ*degP*	BW25113, *degP::kan*, Kan^R^	Keio Collection, [35]
**Plasmids**		
pASK-IBA2	Expression vector with AHTC inducible promoter, Amp^R^	IBA technologies
pASK-IBA2_*invA*	*invA* gene in XbaI-HindIII sites of pASK-IBA2, Amp^R^	This study
pASK-IBA2_*invA*-HA derivatives	Inv HA40, Inv HA204, Inv HA300, Inv HA325 Δ326–835 in XbaI-HindIII sitesof pASK-IBA2, Amp^R^	This study
pASK-IBA2_*eaeA*	*eaeA* gene in XbaI-HindIII sites of pASK-IBA2, Amp^R^	This study
pASK-IBA2_*eaeA*-HA derivatives	Int HA46, Int HA325, Int HA416, Int HA433 Δ434–939 in XbaI-HindIII sitesof pASK-IBA2, Amp^R^	This study
pET28a	Expression vector with IPTG inducible T7 promoter, Kan^R^	Novagen
pET28a_InvMA	Transmembrane domain with alpha-helical linker (AS 93–325) of *invA* inNcoI-HindIII sites of pET28a, Kan^R^	This study
pBAD28_*surA*	*E.c. surA* gene in SacI-HindIII sites of pBAD28	This study

### Site-directed Mutagenesis

To exchange the highly conserved amino acid glycine (G260) in the invasin beta-barrel, site directed mutagenesis was used. A pair of complementary primers both including the appropriate nucleotide sequence to yield the desired amino acid at position G260 were used for PCR. Plasmid pASK-IBA2_*invA* was used as a template. The derived PCR product was directly used for transformation into competent *E. coli* BL21omp2. Correct sequence of the resulting plasmid was verified.

### Generation of Invasin and Intimin Constructs

To introduce two linked HA-epitopes (GSGYPYDVPDYAGSGYPYDVPDYAGSG) into *Yersinia enterocolitica* serotype O:8 derived invasin and EPEC O127:H6 strain 2348/69 derived intimin two PCR reactions were performed using the PfuUltra™ II Fusion HS DNA Polymerase (Stratagene, La Jolla, California). Sequences of all primers that were used are available upon request. In a first round of amplification pASK-IBA2_*invA* or pASK-IBA2_*eaeA* were used as templates to amplify the 5′ part of the coding sequence with the HA tag at its 3′ end and the 3′ part with the HA tag at its 5′ end. The resulting PCR products have overlapping regions that allow hybridization and subsequently the fusion of the two pieces. PCR reactions were purified by gel extraction, mixed in a 1∶1 ratio and then used as templates for the final PCR reaction. The tagged variants of *invA* and *eaeA* were then cloned into pASK-IBA2 expression vector like the wild type genes. Subsequently, all mutants were verified by DNA sequencing. To experimentally prove the correctness of our domain border prediction we amplified the membrane anchor domain plus the alpha-helical linker (AS 93–325) of *Yersinia* invasin with an N-terminal 6xHis-Tag using pASK-IBA2_*invA* as template DNA by PCR. After digestion with NheI and HindIII the PCR product was introduced into pET28a expression vector (Novagen, Darmstadt, Germany) under the control of an IPTG-inducible T7 promoter.

### Protein Production and Purification

Membrane anchor constructs of invasin were produced as cytoplasmic inclusion bodies in the strain BL21(DE3) at 37°C in lysogeny broth (LB) medium [48]. Cells were harvested by centrifugation after 2 h of induction with 1 mM isopropyl-β-D-thiogalactoside (IPTG). Cells were disrupted using a French Press, and inclusion bodies were harvested by differential centrifugation [23]. Inclusion bodies were solubilized using a buffer based on 6 M Guanidine-HCl and were subjected to denaturing Ni-NTA affinity chromatography. The collected fractions were checked for purity using SDS-PAGE - to this end, 20 µl of each fraction were precipitated using 90% ice-cold ethanol to remove the guanidine - and pure invasin fractions were pooled. For refolding, different detergents were screened [49], and LDAO was found to be the most efficient. For large-scale refolding, the guanidine-containing protein solution was quickly diluted 1∶10 into refolding buffer 1% LDAO, 10% Glycerol, 20 mM 3-(N-morpholino)propanesulfonic acid (MOPS)-NaOH pH 7. Subsequent dialysis against the same buffer ensured complete removal of residual guanidine and imidazole.

### Gel Shifts

Heat modifiability, also referred to as gel shifts, is an established method to assay the folding state of β-barrel proteins [23]. Samples were heated to 95°C in standard SDS sample buffer for 10 min, and compared to unheated samples in the same buffer, using a normal SDS-PAGE system (12% gels, Acrylamide/Bisacrylamide ratio 37.5∶1).

### CD Spectroscopy

CD spectra were recorded on a Jasco J-810 spectrophotometer in a 0.1 mm quartz cuvette. 10 spectra were accumulated per measurement, using a data pitch of 0.1 nm, scan speed 20 nm/s, 1 nm slit width and a response time of 2 s. 0.15 mg/ml of refolded protein in 1% LDAO, 10% Glycerol, and 20 mM MOPS-NaOH pH 7 was used in all measurements.

### Bacterial Strains and Growth Conditions

The bacterial strains used in this study are summarized in Table 1. *E. coli* BL21omp2 was transformed with pASK-IBA2 expression vectors containing wild-type or mutant *invA* or *eaeA,* respectively. The strains were grown at 37°C in soy broth supplemented with a piece of autoclaved bovine liver and 100 µg/ml ampicillin. Overnight cultures were diluted into fresh medium to an OD_600_ of 0.05. Bacterial cultures were then subcultured for 2 h at 37°C before anhydrotetracycline (AHTC; IBA GmbH, Göttingen, Germany) was added to a final concentration of 200 ng/ml. If not stated otherwise, bacteria were then allowed to express wild-type or mutant invasin or intimin for another 2 h.

### Sample Preparation for Western Blot Analysis

For preparation of whole-cell lysates, bacterial pellets were resuspended in SDS sample buffer to obtain 5×10^6^ bacteria per ml and incubated for 10 min at 95°C. In order to assess the stability of wild-type or mutant invasin or intimin, urea was added to whole-cell lysates to a final concentration of 2 M and the samples were incubated for 10 min at 85°C.

### Western Blot Analysis

Proteins resolved by SDS-PAGE were transferred onto nitrocellulose membranes. The membranes were blocked overnight with TBS/T (5 mM Tris-HCl, 138 mM NaCl, 0.1% Tween-20, pH 8.0), 3% bovine serum albumine (BSA) (w/v) at 4°C. Blots were probed with purified IgG fraction of polyclonal rabbit anti-InvA (1∶5000), anti-EaeA (1∶1000), anti-BamA (1∶5000), anti-SurA (1∶10 000), anti-Skp (1∶5000), anti-GyrA (1∶1000; Inspiralis, Norwich, UK), anti-groEL (1∶1000), guinea pig anti-DegP (1∶5000) or monoclonal mouse anti-HA (Santa Cruz Biotechnology, Santa Cruz, USA) antibodies and a peroxidase-conjugated secondary anti-rabbit (diluted 1∶10 000; Dianova, Hamburg, Germany) anti-guinea pig (diluted 1∶5000; Jackson ImmunoResearch, Pennsylvania, United States) or anti-mouse antibody (diluted 1∶1000; Dako, Denmark). Anti-BamA, -InvA and -EaeA sera had been pre-adsorbed against paraformaldehyde (PFA)-fixed bacteria deficient for the respective antigen before.

### Preparation of Outer Membrane Fractions

Preparation of outer membranes was carried out using 50 ml bacterial culture. Cells were harvested and resuspended in 500 µl resuspension buffer (0.2 M Tris, 1 M Sucrose, 1 mM EDTA, pH 8). After addition of 500 µg lysozyme (20 U/µg) and 3.2 ml water the samples were incubated for 20 min at room temperature. Protoplasts were lysed in 5 ml lysis buffer (2% Triton X-100, 50 mM Tris, 10 mM MgCl_2_, pH 8) and released DNA was digested by addition of 50 µg DNAse I (10 mg/ml, Roche, Mannheim, Germany). Outer membranes were pelleted by centrifugation at 85,000× *g* for 30 min at 4°C. After 3 washing steps with water the membranes were resuspended in SDS sample buffer.

### Urea Extraction of Outer Membrane Preparations

Bacterial envelopes were pelleted by ultracentrifugation at 290,000× *g* for 1 h. The membranes were resuspended in 1 ml urea extraction solution (100 mM glycine, 6 M urea, 15 mM Tris-HCl, pH 7,4) and extracted for 1 h at 37°C. Membranes from urea-treated samples were reisolated by centrifugation at 290,000× *g* for 90 min at 25°C and resuspended in SDS sample buffer.

### Immunofluorescence Microscopy

For immunofluorescence stainings 1×10^7^ bacteria in PBS were centrifuged on polyethyleneimine-coated coverslips, fixed for 30 min with 4% PFA in PBS (w/v) and subsequently blocked with 1% bovine serum albumin (BSA) in PBS (w/v) at room temperature. For stainings of periplasmic HA tags, bacterial cell walls were permeabilized for 20 min in 0.5% Triton X-100/PBS (v/v). Stainings were performed using preadsorbed polyclonal rabbit antibody directed against InvA (diluted 1∶200) or EaeA (diluted 1∶50) and a 1∶200 dilution of a Cy2-conjugated secondary anti-rabbit antibody (Dianova, Hamburg, Germany). HA tags were stained with monoclonal mouse antibodies (diluted 1∶200) and Cy3-conjugated secondary anti-mouse antibody (diluted 1∶200, Dianova, Hamburg, Germany). Secondary antibodies were incubated at room temperature for 2 h in a dark chamber. Bacterial DNA was stained with 0.01 mg/ml 4′,6-diamidino-2-phenylindole (DAPI). Finally, coverslips were mounted with Mowiol (Merck, Darmstadt, Germany). Fluorescence images were obtained with a DMRE fluorescence microscope (Leica, Wetzlar, Germany).

### Quantification of Invasin Surface Localisation by Flow Cytometry

Protein production in bacteria was induced with AHTC to a final concentration of 200 ng/ml. After 2 h of induction, 5×10^7^ bacteria were harvested by centrifugation. Cells were washed once with PBS, fixed with 4% PFA, washed again, blocked with 1% BSA and after washing resuspended in PBS. Samples were stained with a purified IgG fraction of the rabbit anti-InvA (1∶200) antibody and Cy2-conjugated secondary antibody (1∶200; Dianova) for 2 h at room temperature and then washed twice with PBS. Surface localization of invasin was measured by flow cytometry using a LSRFortessa cell analyzer (Becton Dickinson, Heidelberg, Germany), and data were analyzed with WinMDI (J. Trotter) software. The results of one representative experiment are shown.

### Cell Culture and IL-8 ELISA

HeLa cells (ATCC number: CCL-2) were cultivated in RPMI-1640 (Biochrom, Berlin, Germany) medium supplemented with 2 mM L-glutamine (Sigma, Deisenhofen, Germany) and 10% fetal calf serum (FCS; Gibco, Darmstadt, Germany) (experiments were also performed with serum-starved cells and gave comparable results). Infection using a multiplicity of infection (MOI) of 100 was carried out as described for the adhesion assay with invasin-producing bacteria. After 1 h of infection, extracellular bacteria were killed by the addition of gentamicin (100 µg/ml) and the cell cultures were incubated for 5 more hours. The cell culture supernatant was collected and the IL-8 ELISA was carried out as described previously [50],. IL-8 concentrations were calculated using recombinant human IL-8 (Becton Dickinson, Heidelberg, Germany ) as a standard.

### Intimin Adhesion Assay and SEM Analysis

1.5×10^5^ HeLa cells were seeded onto coverslips and grown overnight in Dulbecco’s modified Eagle’s medium (DMEM; PAA, Pasching, Austria) supplemented with 10% fetal calf serum (FCS), 1% L-glutamine and 1% penicillin/streptomycin (Pen/Strep; Gibco, Darmstadt, Germany). The next day, cells were washed twice and incubated again at 37°C and 5% CO_2_ for one hour before preinfection with the E2348/69 Δ*eaeA* EPEC strain. Cells were preinfected in order to prime HeLa cells with the Tir receptor necessary for the binding of intimin. Overnight cultures of the EPEC *eaeA* mutant strain were subcultivated for 2 h at 37°C, harvested by centrifugation (4000× *g*, 5 min) and washed once with PBS. Then, HeLa cells were infected at a MOI of 20. Bacteria were centrifuged onto the cells at 500× *g* for 1 min and incubated for 3 h at 37°C, 5% CO_2_ followed by 4 washing steps. Remaining adherent bacteria were killed by incubation with gentamicin (100 µg/ml) for 1 h. Finally, cells were washed with medium without antibiotics. For infection with *E. coli* omp2 strains producing the wild-type and HA-tagged versions of intimin, bacteria were subcultivated as described above. After 3 hours of protein production, bacteria were harvested and washed once with PBS. The preinfected HeLa cells were then infected at a MOI value of 20 for 2 h. Following 3 washing steps with PBS, the cells were fixed overnight with 4% PFA in PBS. Afterwards, cells were permeabilized with 0.1% Triton X-100 in PBS for 10 min. To be able to distinguish between possibly adherent EPEC *eaeA* mutant bacteria and the intimin-producing *E. coli*, the permeabilized cells were stained with anti-intimin antibodies. Finally, the coverslips were mounted in Mowiol and analysed with a fluorescent microscope with appropriate filter settings. For preparation of samples for SEM analysis cells were infected and fixed with PFA as described above. Afterwards, the preparation was essentially carried out as described [51] and analyzed in a Hitachi S-800 field emission scanning electron microscope.

### Invasin Adhesion Assay

1×10^5^ human HeLa cells per well were seeded on coverslips in a 24-well microplate and grown overnight in RPMI-1640 supplemented with 10% FCS, 2 mM L-glutamine and Pen/Strep. The next day, cells were washed once with prewarmed PBS and grown for another 1 hour in RPMI-1640 10% FCS without antibiotics. Two wells were trypsinized (Gibco, Darmstadt, Germany) and the number of cells per well was determined. Bacteria grown overnight at 37°C were diluted to OD_600_ 0.05 into fresh medium with antibiotics and grown for 2 h at 37°C. Then, production of invasin was induced by addition of AHTC (final concentration 200 ng/ml) for 2 h. Afterwards bacteria were harvested by centrifugation (5 min, 4000× *g* in a tabletop centrifuge) and washed once with prewarmed PBS. OD_600_ was determined and the HeLa cells were infected with bacteria with a MOI of 100. Bacteria were spun down on the cells by a short centrifugation step (1 min, 500× *g*) and incubated for 1 h at 37°C. Non-adhering bacteria were removed by 3 washing steps with prewarmed PBS. HeLa cells with the attached bacteria were fixed with 4% PFA in PBS for 10 min at room temperature. The fixed samples were washed once with PBS and stained with an aqueous solution of fuchsine for 2 minutes. The stained samples were washed once in PBS dipped shortly in distilled water and air dried. Finally the coverslips were mounted in Entellan (Merck, Darmstadt, Germany) and examined with a light microscope. Pictures of randomly chosen areas were taken at a 100-fold magnification.

### Testing BamA and Chaperone Dependency of Invasin Biogenesis

*E. coli* K-12 derivative MC4100A and the corresponding BamA depletion strain were transformed with pASK-IBA2 expression vector containing wild-type *invA*. The BamA mutant strain was supplied with 0.2% glucose (w/v) for depletion or 0.2% arabinose (w/v) for rescue of BamA production and with 50µg/ml kanamycin. Overnight cultures were washed twice and resuspended in fresh medium to an OD_600_ 0.05. To test presence of invasin under depletion conditions of BamA, whole-cell lysates were prepared before induction of *invA* expression and also 60 min and 120 min after induction. To investigate the role of the periplasmic chaperones Skp, DegP and SurA in the biogenesis of invasin, pASK-IBA2_*invA* was transformed into *E. coli* BW25113 and the corresponding single knockout mutant strains (National BioResource Project; NIG, Japan). 2h after starting protein production, whole-cell lysates of invasin-producing bacteria were prepared and InvA levels as well as the phenotype of the *E.coli* BW25113 knock out strains were analyzed on Western blots, flow cytometry and immunofluorescence stainings. For complementation of the *surA* knockout strain, plasmid pBAD28_*surA* was transformed into *E.coli* BW25113 Δ*surA* and 0.2% arabinose (w/v) as well as 50 µg/ml chloramphenicol were added to the growth medium.

## Supporting Information

Figure S1 **Alignment if intimin/invasin family proteins, reproduced at a higher resolution from** **Fig. 1A** **.** The amino acid sequences of the N-terminal part of each protein (excluding the extracellular passenger domain) were analysed using SignalP, PsiBLAST and HHAlign. α-helical segments (magenta) and β-strands (blue) are indicated by different colouring. The sequences are mainly from enterobacteria; gi numbers are provided.
(TIF)

Figure S2 **Intimin but not invasin shows dimerisation after crosslinking with BS3.** Outer membranes of E.coli BL21(DE3)omp2 expressing wild-type intimin, wild-type invasin or invasin G260 mutants were prepared and incubated with (+) or without (–) the crosslinker BS3 (Thermo Scientific) according to the manufacturer’s instructions. The samples were subjected to SDS-PAGE and western blots were probed with antibodies against invasin or intimin.
(TIF)
