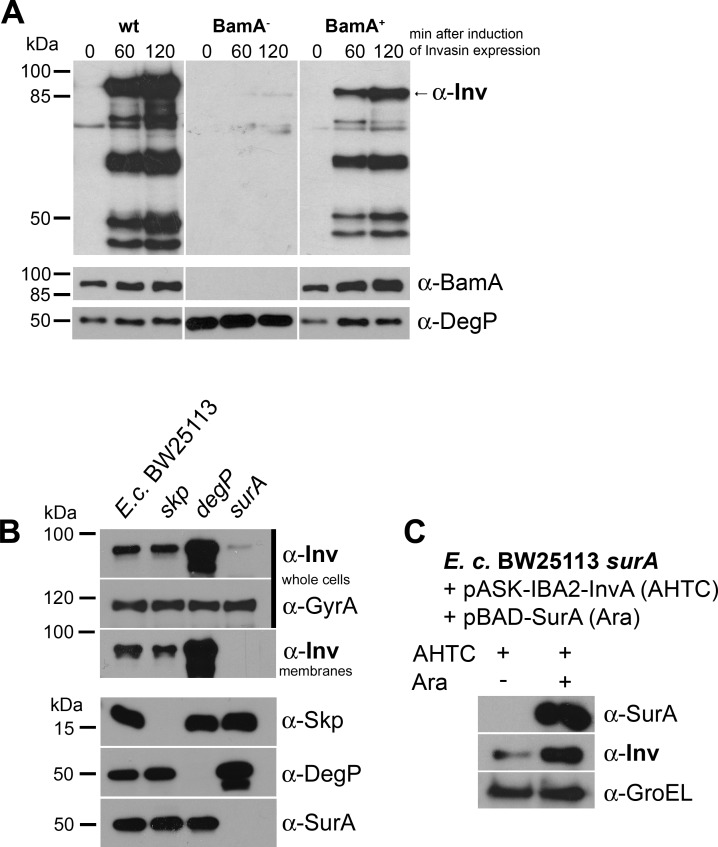# Correction: Intimin and Invasin Export Their C-Terminus to the Bacterial Cell Surface Using an Inverse Mechanism Compared to Classical Autotransport

**DOI:** 10.1371/annotation/cb7e47be-dd4b-46cd-b4e5-18b7077f64df

**Published:** 2012-11-06

**Authors:** Philipp Oberhettinger, Monika Schütz, Jack C. Leo, Nadja Heinz, Jürgen Berger, Ingo B. Autenrieth, Dirk Linke

There was an error in Figure 8. The correct figure can be found here: 

**Figure:**