# Supplementary figures and images for: Intimin and Invasin Export Their C-Terminus to the Bacterial Cell Surface Using an Inverse Mechanism Compared to Classical Autotransport
Source: PLoS One. 2012 Oct 9;7(10):e47069. doi: 10.1371/journal.pone.0047069 (PMC3467248; doi:10.1371/journal.pone.0047069)

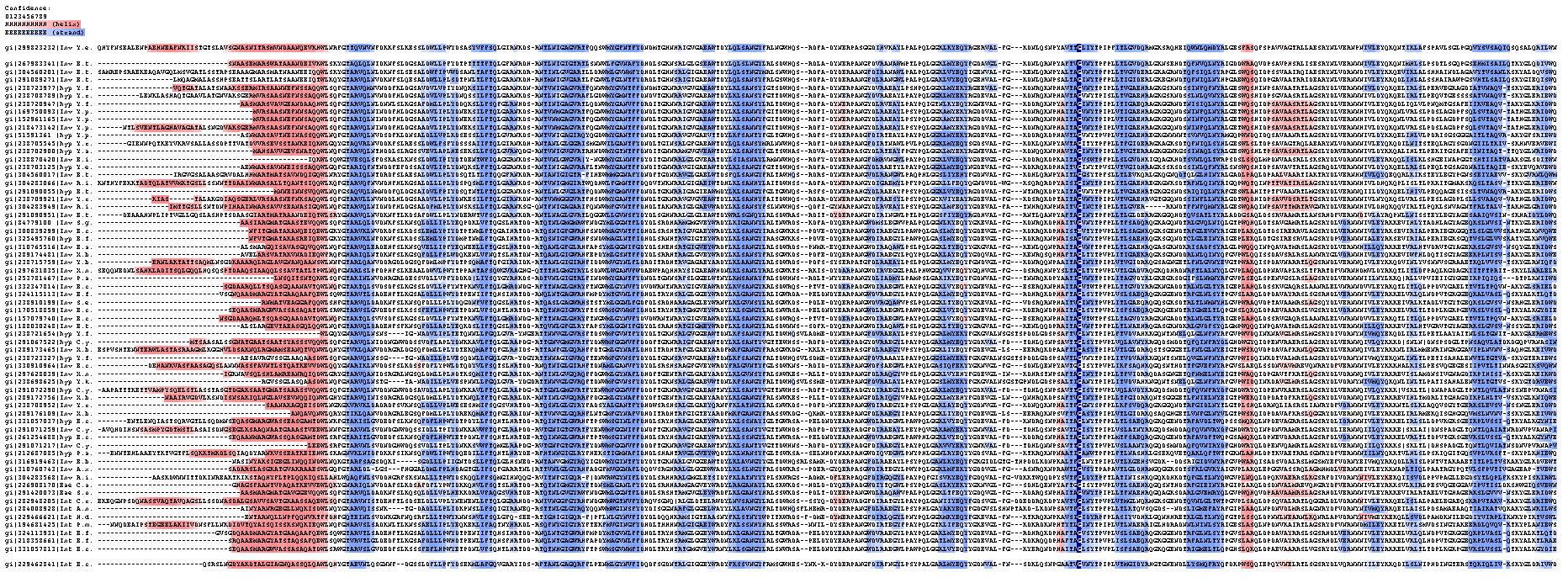

Supplement: Figure S1 — Alignment if intimin/invasin family proteins, reproduced at a higher resolution from Fig. 1A . The amino acid sequences of the N-terminal part of each protein (excluding the extracellular passenger domain) were analysed using SignalP, PsiBLAST and HHAlign. α-helical segments (magenta) and β-strands (blue) are indicated by different colouring. The sequences are mainly from enterobacteria; gi numbers are provided. (TIF) [file pone.0047069.s001.tif]

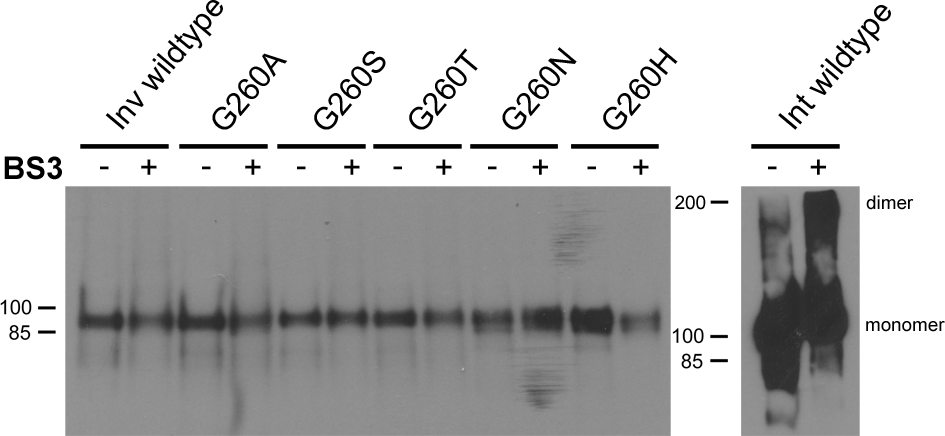

Supplement: Figure S2 — Intimin but not invasin shows dimerisation after crosslinking with BS3. Outer membranes of E.coli BL21(DE3)omp2 expressing wild-type intimin, wild-type invasin or invasin G260 mutants were prepared and incubated with (+) or without (–) the crosslinker BS3 (Thermo Scientific) according to the manufacturer’s instructions. The samples were subjected to SDS-PAGE and western blots were probed with antibodies against invasin or intimin. (TIF) [file pone.0047069.s002.tif]
